# WHO MAINTAINS GOOD HEALTH? CONTRIBUTIONS OF BEHAVIORS AND LIFE COURSE CIRCUMSTANCES TO HEALTHY AGING

**DOI:** 10.1093/geroni/igae098.1784

**Published:** 2024-12-31

**Authors:** Chenlu Hong, Yanan Luo

**Affiliations:** Peking University, Beijing, Beijing, China (People’s Republic); Peking University, Beijing, Beijing, China (People’s Republic)

## Abstract

**Background:**

Life course circumstances, encompassing diverse environmental conditions and experiences throughout an individual’s life, are critical determinants of healthy aging. However, limited evidence focuses on their cumulative effects on various aging trajectories and disparities among different subpopulations. Using the healthy ageing scores (HAS), this study aimed to evaluate the relative contributions of life course circumstances in childhood and adulthood to healthy aging among Chinese older adults.

**Methods:**

Utilizing data from the China Health and Retirement Longitudinal Study (CHALRS), we assembled for 7906 middle-aged and older adults in China. HAS, developed by the Ageing Trajectories of Health Longitudinal Opportunities and Synergies (ATHLOS), was calculated to measure overall biopsychosocial aspects of health and functioning, with higher values indicating better health maintenance in later life. Behavioral factors included smoking, drinking and obesity. Life course factors encompassed socioenvironmental circumstances in childhood and adulthood. Linear regression models were employed to analyze the role of life course circumstances domains in healthy aging and estimate the associations of identified subpopulations with healthy aging. The ShapleyValue Decomposition, principal components analysis and hierarchal cluster analysis were performed.

**Results:**

We identified four subpopulations sharing similar life courses characterized by disadvantaged or advantaged childhood and adulthood circumstances. Of these, the most disadvantaged subpopulation, experiencing more adulthood adversity and childhood trauma, along with worse adulthood socioeconomic status, consistently exhibited the lowest levels of healthy aging and coversely the most advantaged subpopulation, characterized by better adulthood SES and childhood SES, demonstrated higher Healthy Aging Scores.

